# Auditory Scene Analysis Deficits Across Dementia Subtypes: A Framework for Environmental Design

**DOI:** 10.1002/alz70858_098774

**Published:** 2025-12-25

**Authors:** Arezoo Talebzadeh, Dick Botteldooren

**Affiliations:** ^1^ University Ghent, Ghent, Flanders, Belgium

## Abstract

**Background:**

Auditory Scene Analysis (ASA), the brain's ability to organize and interpret complex acoustic environments (Bragman, 1993), is differentially impaired across dementia subtypes. While research has established the impact of soundscape on behavioural and psychological symptoms of dementia (BPSD) (Janus et al., 2021; De Pessemier et al., 2022; Talebzadeh, Decoutere et al., 2023; Talebzadeh et al., 2024) limited work has examined how distinct ASA deficits in different forms of dementia should inform environmental design. This study synthesizes clinical trials and observational studies findings to develop a theoretical framework linking dementia‐specific ASA impairments to targeted soundscape interventions.

**Methods:**

We conducted a systematic analysis of ASA deficits across dementia subtypes through multiple complementary approaches. First, we performed ethnographic observations in dementia care settings to understand the relationship between acoustic environments and behavioural responses. These observations informed the development of a randomized controlled trial (*n* = 28) examining soundscape augmentation effects on BPSD. Finally, we synthesized these findings through functional block diagrams mapping the relationship between auditory deficits and dementia variants, integrating both the theoretical ASA framework and empirical intervention outcomes.

**Results:**

Analysis revealed distinct patterns of ASA impairment across dementia subtypes: Alzheimer's disease showed primary deficits in sound source segregation and spatial processing; posterior cortical atrophy demonstrated severe ASA segregation/grouping impairments exceeding typical Alzheimer's; frontotemporal dementia exhibited specific deficits in rhythm, pitch, and emotional processing; while Lewy body dementia showed disrupted reality monitoring and auditory hallucinations. The RCT demonstrated that soundscape augmentation could improve behavioural symptoms, with significant reductions in resistance to care scores (‐0.81, 95% CI ‐1.59 to ‐0.03, *p* = 0.042), though these improvements were not specifically linked to dementia subtypes. These findings, combined with the identified ASA deficit patterns, suggest the potential for more targeted interventions.

**Conclusions:**

This work establishes a novel framework linking dementia‐specific ASA deficits to environmental design principles. Results indicate that soundscape interventions must be tailored to the particular pattern of auditory processing impairment in different dementia subtypes. These findings have significant implications for designing acoustic environments in dementia care settings and suggest that environmental modifications should consider both bottom‐up (primitive) and top‐down (schema‐based) auditory processing deficits specific to each form of dementia.

